# PaintOmics 4: new tools for the integrative analysis of multi-omics datasets supported by multiple pathway databases

**DOI:** 10.1093/nar/gkac352

**Published:** 2022-05-24

**Authors:** Tianyuan Liu, Pedro Salguero, Marko Petek, Carlos Martinez-Mira, Leandro Balzano-Nogueira, Živa Ramšak, Lauren McIntyre, Kristina Gruden, Sonia Tarazona, Ana Conesa

**Affiliations:** Department of Mechanical Engineering, School of Engineering, Cardiff University, Cardiff, UK; Department of Applied Statistics, Operations Research and Quality, Universitat Politècnica de València, Valencia, Spain; Department of Biotechnology and Systems Biology, National Institute of Biology, Ljubljana, Slovenia; Biobam Bioinformatics, Valencia, Spain; Diabetes Institute, University of Florida, Gainesville, USA; Department of Biotechnology and Systems Biology, National Institute of Biology, Ljubljana, Slovenia; Department of Molecular Genetics and Microbiology, Genetics Institute, University of Florida, Gainesville, USA; Department of Biotechnology and Systems Biology, National Institute of Biology, Ljubljana, Slovenia; Department of Applied Statistics, Operations Research and Quality, Universitat Politècnica de València, Valencia, Spain; Institute for Integrative Systems Biology, Spanish National Research Council (CSIC), Paterna, Spain; Department of Microbiology and Cell Science, University of Florida, Gainesville, FL, USA

## Abstract

PaintOmics is a web server for the integrative analysis and visualisation of multi-omics datasets using biological pathway maps. PaintOmics 4 has several notable updates that improve and extend analyses. Three pathway databases are now supported: KEGG, Reactome and MapMan, providing more comprehensive pathway knowledge for animals and plants. New metabolite analysis methods fill gaps in traditional pathway-based enrichment methods. The metabolite hub analysis selects compounds with a high number of significant genes in their neighbouring network, suggesting regulation by gene expression changes. The metabolite class activity analysis tests the hypothesis that a metabolic class has a higher-than-expected proportion of significant elements, indicating that these compounds are regulated in the experiment. Finally, PaintOmics 4 includes a regulatory omics module to analyse the contribution of trans-regulatory layers (microRNA and transcription factors, RNA-binding proteins) to regulate pathways. We show the performance of PaintOmics 4 on both mouse and plant data to highlight how these new analysis features provide novel insights into regulatory biology. PaintOmics 4 is available at https://paintomics.org/.

## Graphical Abstract

**Graphical Abstract:**
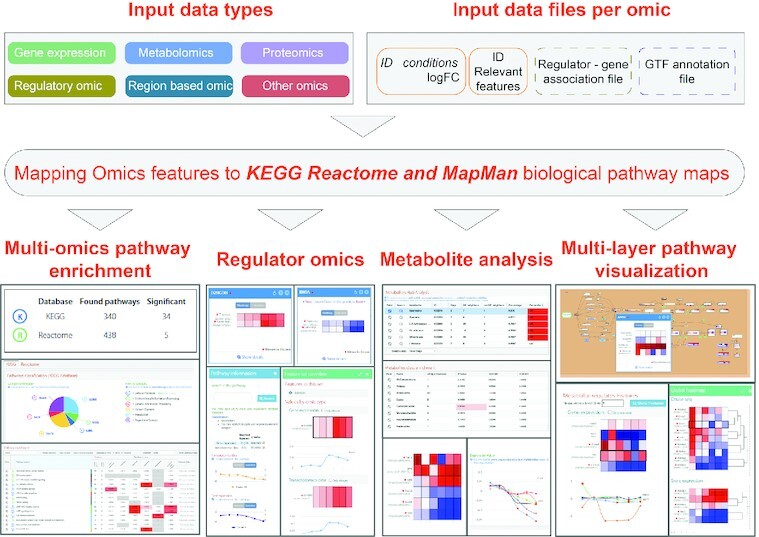
Overview of Paintomics 4 functionalities.

## INTRODUCTION

Multi-omics approaches have become popular in the study of a wide range of biological domains, with multi-omics datasets being now commonly obtained by individual investigators as well as large consortia (1–3). Moreover, the number and diversity of measured omics modalities have also increased, with former studies combining at most two or three omics platforms, and more recently genomics, transcriptomics, methylomics, chromatin accessibility, proteomics, metabolomics and/or lipidomics often combined in a single study (4–6). Multiple approaches have been proposed for the integrative analysis of these data (see (7,8) for reviews). Current methods can be broadly divided into three groups: detecting biomarkers, classifying samples, and inferring functional relationships between molecular layers (9). Regardless of the aim of these analyses, the subsequent biological interpretation of the analysis results is frequently difficult and time-consuming, as it requires human interaction and comprehension. Biological interpretation of multi-omics models is a grand challenge faced by investigators interrogating multi-omics data (9).

Three major strategies may be applied for the interpretation of multi-omics data. Overrepresentation and enrichment analyses are widely used in genomics and transcriptomics analyses (10). These methodologies have been adapted to the different omics data types (11–13), as well as to their integrative analysis (14–16). Enrichment methods are powerful tools to identify which biological processes are regulated in a given condition, but they are limited by the vocabulary, lack of comprehensive annotations and absence of mechanistic insight within and across omics layers.

An alternative approach is to use multi-partite networks, where nodes depict molecular entities and edges indicate regulatory or covariance information that can be extracted from the integrative statistical analysis (17). A variety of network (graphical) resources (e.g. Cytoscape (18), 3Omics (19), VisANT (20), OmicsAnalyst (21)) may be used to visualise relationships among biomolecules. Unfortunately, networks are frequently too large and interpretability is limited by the lack of context. A recent novel related method is multiSlide, which visualizes interconnected molecular features in heatmaps of multi-omics data sets (22).

A third option is to leverage existing biological knowledge represented in pathway maps to project multi-omics data and visualise them within a highly interpretable format. Examples are kaPPA-view 4 (23), MapMan4 (24) and PaintOmics 3 (14). Some tools may include several of these options. For instance, Cytoscape, OmicsAnalyst and Paintomics 3 also support enrichment analysis.

Pathway-based visualization methods also have limitations. First, they lack the flexibility to incorporate significant features suggested by the statistical analysis since absent in the available map. Moreover, interpretation is limited by the pathway boundaries decided by curators and by the amount and identity of the molecular features captured in the maps (25). Some of the measured features may not have a pathway location and some pathway components might not be measured. Additionally, some pathway elements might correspond to multiple measured features, such as a protein complex or a gene family. The limitations of pathway methods are particularly evident in untargeted and semi-targeted metabolomics data, since many measured compounds are unidentified, or only identified based on a large class of similar compounds (i.e. lipids) and therefore not present on current maps. Consequently, pathway-based enrichment methods behave poorly on metabolomics data and other strategies, such as metabolite-class enrichment (26) might be more appropriate. Here we present PaintOmics 4, a substantial expansion of the Paintomics 3 web server for pathway-based multi-omics data analysis (14,27), that addresses some of these current limitations in pathway definitions, metabolomics data integration and visualisation across molecular layers. An overview of the new functionalities is provided in Supplementary Figure S1.

## MATERIALS AND METHODS

### Implementation of Reactome and MapMan pathway databases into PaintOmics 4

Next to KEGG, PaintOmics 4 adds Reactome and MapMan to the list of pathway databases supported by the application (Supplementary Figure S2) (24,28). The new pathway data was incorporated into the existing PaintOmics MongoDB where pathways are classified into categories and features are mapped to the lowest pathway level. All PaintOmics data structures were modified to include a ‘source database’ field. This resulted in 904 new pathways distributed into 29 categories from Reactome and 25 new pathways in two categories from MapMan added to PaintOmics 4. Queries to the integrated database now proceed in batches to accommodate the larger number of entities to be searched. PaintOmics represents multi-omics data on pathways by overlaying feature expression and intensity values on their box positions in the pathway map using a colour scheme. In order to display multi-omics data on Reactome pathways, node coordinates available in pathway XML files were used. Since MapMan BIN coordinates are approximate, all ManMap pathway images were manually inspected and when required, XML files were edited to ensure the correct painting of data. Currently, Paintomics 4 implements KEGG Release 102.0 April 1 2022, Reactome Version 76 21 March 2021 and GoMapMan 25 May 2018. Databases are updated annually.

### Regulatory Omics functionality

PaintOmics 3 provided the Regulatory Omics option designed to upload data on features such as microRNA-seq, acting as regulators of gene expression. PaintOmics 4 extends this functionality to accept any type of trans-acting element operating on genes, transcripts or proteins and includes filtering functions to extract meaningful regulatory relationships. In addition to microRNA-seq, transcription factors (TF) and splicing factors (SF), detected by RNA-seq, RNA-binding proteins identified by CLIP-seq, etc., can be analysed with this option. The Regulatory Omics option takes a trans-regulatory-feature data matrix with expression or activity values for regulators in the conditions of the study. The regulator-gene/protein mapping file is provided by the user, together with an optional list of significant deferentially expressed regulators. PaintOmics 4 filtering options include thresholds for positive or negative correlation to select the expected regulatory relationships. Applying these criteria, regulatory features will be mapped to their targeted features and their corresponding pathways. A pathway enrichment score is calculated either based on the number of regulators mapping to each pathway or on the number of regulated genes present in the pathway. Enriched pathways for the Regulatory Omics modality represent biological processes that are significantly impacted by that regulatory layer.

### Novel metabolomics interpretation methods

#### Metabolite hub analysis

**Figure 1.**
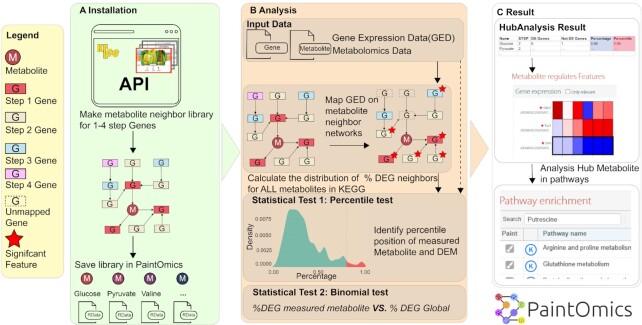
New metabolomics analysis in PaintOmics 4. (**A**) Neighbouring genes for each metabolite at 1 to 4 network steps are identified. (**B**) The percentile and binomial tests are used to identify metabolites with a high density of DEGs in their proximal network. (**C**) Metabolites and Genes identified in the analysis are shown as heatmaps with links to associated pathways. DEM: differentially expressed metabolite; DEG: differentially expressed gene.

One of the goals of multi-omics studies that combine metabolomics and gene expression or proteomics data is to associate changes in metabolite levels with the regulation of the enzymes that may contribute to metabolite turnover. PaintOmics 4 leverages pathway information to identify metabolites that have a high proportion of differentially expressed features in their close network. Two tests were implemented (Figure 1). The binomial test is used to evaluate for each differentially expressed metabolite (DEM) the hypothesis that, given the overall percentage of differentially expressed genes (DEG) *p_0_* in the dataset, the proportion of DEG linked to the metabolite is significantly higher than *p_0_*. The hub analysis evaluates genes directly connected in the network (step 1) or genes associated with the metabolite through up to 3 intermediate nodes (steps 2 to 4). *P*-values are corrected for multiple testing (29). Alternatively, the distribution in the overall metabolic network of the percentage of neighbouring DEG for metabolite nodes is computed and the percentile position of each measured metabolite in this distribution is calculated. Metabolites with a high percentile value have a higher proportion of connecting DEG than the majority of the metabolites in the database.

#### Metabolite class activity

To test the hypothesis of a metabolite class being regulated, PaintOmics 4 implements a metabolite class activity analysis tool, where a binomial test is used to assess the hypothesis of the proportion of significant compounds in a given measured metabolite class being higher than a user-defined threshold. In case the user does not define an activity threshold, PaintOmics 4 will use the average percentage of significant metabolites as threshold for the null hypothesis. *P*-values are corrected for multiple testing (29). These novel metabolomics analysis tools are provided as a separate tab in the main PaintOmics results panel that includes hyperlinks to facilitate navigation between metabolite data, neighbouring genes and metabolic pathways (Figure 1).

### Metagenes for nodes and pathways

PaintOmics displays omics data on pathways maps by colouring the node position of the omic feature according to its experimental value. When a node contains multiple features, e.g. MapMan BINs, the map topology may not be able to accommodate the amount of data. In order to address this problem, metagenes are computed for pathway nodes with more than four matching features (30), resulting in a compressed representation of omics data in complex nodes that fits available space on the map. Note that when one node contains features with different profiles, the analysis might return multiple metagenes for the node, one per profile type.

### Use case datasets

PaintOmics 4 functionalities were demonstrated using two different multi-omics datasets. The STATegra data that collects multi-omics data for a mouse B-cell differentiation process triggered by the expression of the Ikaros TF (4).

The second dataset corresponds to an *Arabidopsis* study that evaluated the root transcriptional and metabolic profile of a BRL3 overexpressing mutant in drought conditions. BRL3 is a vascular-enriched member of the brassinosteroid family, which was speculated to confer drought tolerance (31).

## RESULTS

### STATegra data analysis with PaintOmics 4 reveals novel regulatory events during B-cell differentiation

We used the STATegra multi-omics dataset describing the differentiation of the murine B3 cell line from a proliferating pre-BI state to differentiated pre-BII (4) to demonstrate PaintOmics 4 functionalities. RNA-seq, micro-RNA-seq, DNase-seq, metabolomics and TF data were available. The dataset contains temporal data for 13 123 genes, 469 microRNAs, 10 272 DNaseq regions, 320 TFs and 60 metabolites, of which 5224, 172, 5099, 180 and 40 features, respectively, were found to have significant differences along the differentiation course (4). Five STATegra omics modalities were run in PaintOmics 4 selecting both KEGG and Reactome as pathway databases. KEGG disease and organismal pathways were excluded from the analysis. Data mapped to a total of 169 KEGG and 439 Reactome pathways, of which 14 and 11, respectively, were found significant by the Fisher combined *P*-value method (32) that jointly considers all omics modalities. The full list of significant pathways is provided in Supplementary Table S1.

#### PaintOmics 4 indicates a multi-layered control of B-cell differentiation

We first analysed the overall patterns of pathway changes across molecular layers using the pathway network analysis in PaintOmics 4 to focus attention on the analysis of our Regulatory Omics types, microRNA and TFs (Supplementary Figure S3). This tool revealed that pathways change during B-cell differentiation according to 2–3 patterns. For gene expression, most metabolic and genetic information pathways were downregulated, while signalling pathways showed both up and down regulation trends. microRNAs associated genes in these pathways tended to be upregulated at late-time points (pre-BII stage) in metabolic and genetic information processing pathways, while upregulation for signalling pathways took place at early time points (pre-BI stage). Finally, TF regulation showed the opposite behaviour, with TFs that bind metabolic and genetic information processing pathways being downregulated as differentiation progresses but upregulated for signalling pathway genes. These results indicate a highly coordinated control of biological pathways during B-cell differentiation, characterized by the transcriptional activation of signalling pathways and the downregulation of metabolic activities, with transcriptional (TF) and post-transcriptional (microRNA) mechanisms contributing to this program.

#### KEGG and Reactome complemented each other in the analysis of STATegra multi-omics data

**Figure 2.**
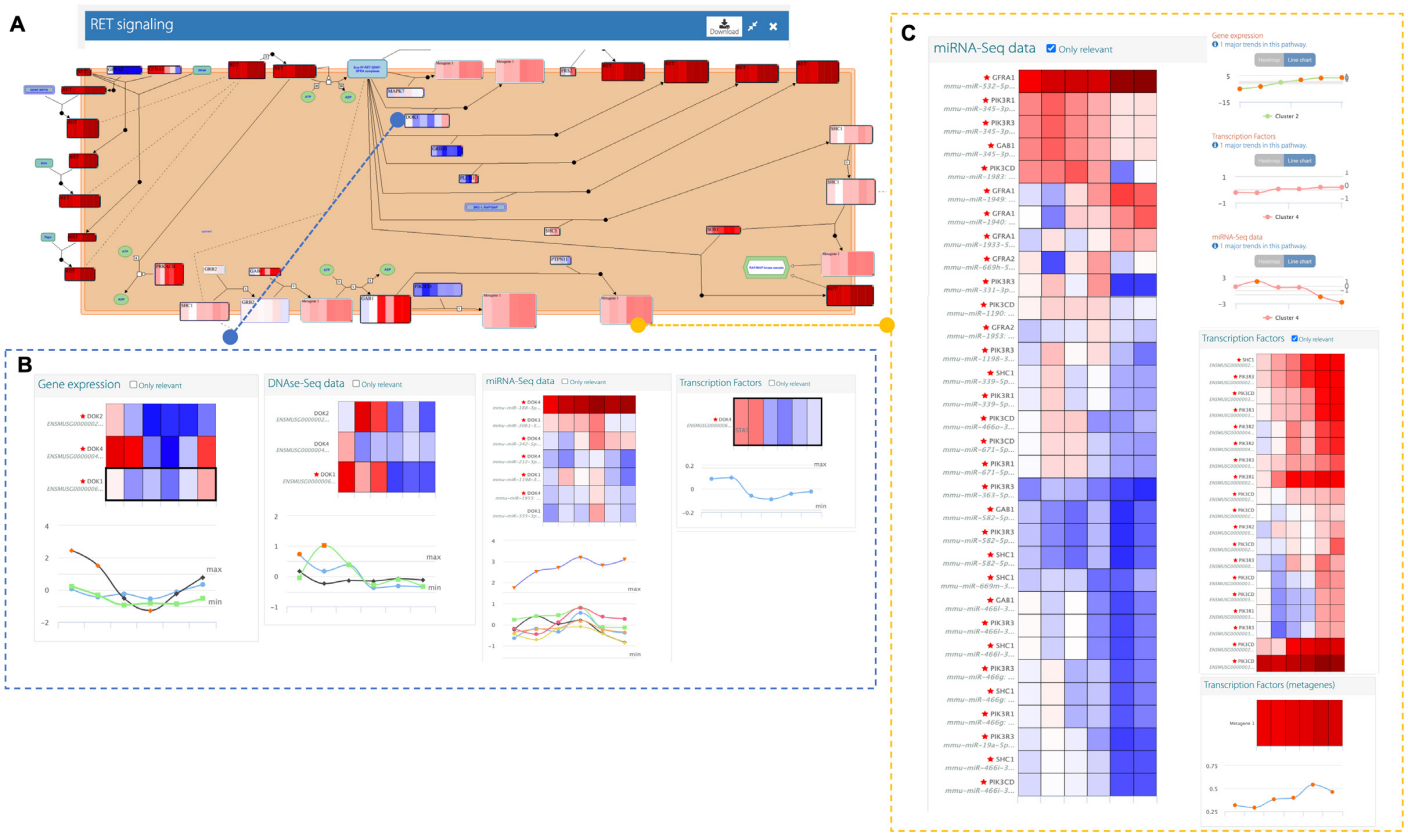
PaintOmics multi-omics analysis of RET signalling pathway. (**A**) RET signalling pathway. (**B**) Multi-omics data for metagene 1 containing receptor proteins. (**C**) Multi-omics data for DOK gene family.

The analysis of enriched KEGG and Reactome pathways indicated commonalities and differences between the two resources (Supplementary Table S1). Both databases reported enrichment of glucose, amino-acids and nucleotide metabolic processes, and of p53 signalling. However, most significant signalling pathways did not coincide, possibly due to different pathway definitions between KEGG and Reactome. The combined analysis revealed many of the known processes operating during the differentiation of the hematopoietic and immune cell lineages, e.g. Interleukin-2 family signalling, Interferon gamma signalling, RAF-independent MAPK1/3 signalling, for Reactome, and JAK-STAT signalling, FOXO signalling, and Hippo signalling for KEGG (33). Reactome but not KEGG identified the RET signalling pathway as enriched (combined *P*-value = 0.029). RET is a tyrosine kinase receptor essential for embryonic development (34) which has also been found to be expressed in hematopoietic tissues, suggesting a role in the development of the immune system. Specifically, RET induces the expression of chemokines and cytokines, and downregulates chemokine/cytokine receptors (35). In the B3 cell differentiation process, we found a strong upregulation of RET and other associated membrane receptors (Figure 2A and B). A concordant regulation by many microRNAs and transcription factors was also found (Figure 2C). Interestingly, a different component of the RET signalling pathway is the DOK protein, represented by three family members (1, 3 and 4) in our data. These genes were downregulated at the transition between pre-BI and pre-BII stages, together with their only significant TF (STAT4: ENSMUSG00000062939), meanwhile the associated microRNA (mir-188-3p) was strongly upregulated. STAT4 is a key TF of the immune cell lineage (36), and mir-188-3p has been reported to regulate cell proliferation (37). Whether these different regulatory relationships in the RET signalling pathway represent specific contributions to the differentiation of B-cells remains to be investigated. Our results showcase the power of PaintOmics 4 pathway-based multi-omics analysis to present and dissect a diversity of multi-layered regulatory relationships.

#### PaintOmics 4 novel metabolomics analysis tools highlight metabolite roles in B-cell differentiation

**Figure 3.**
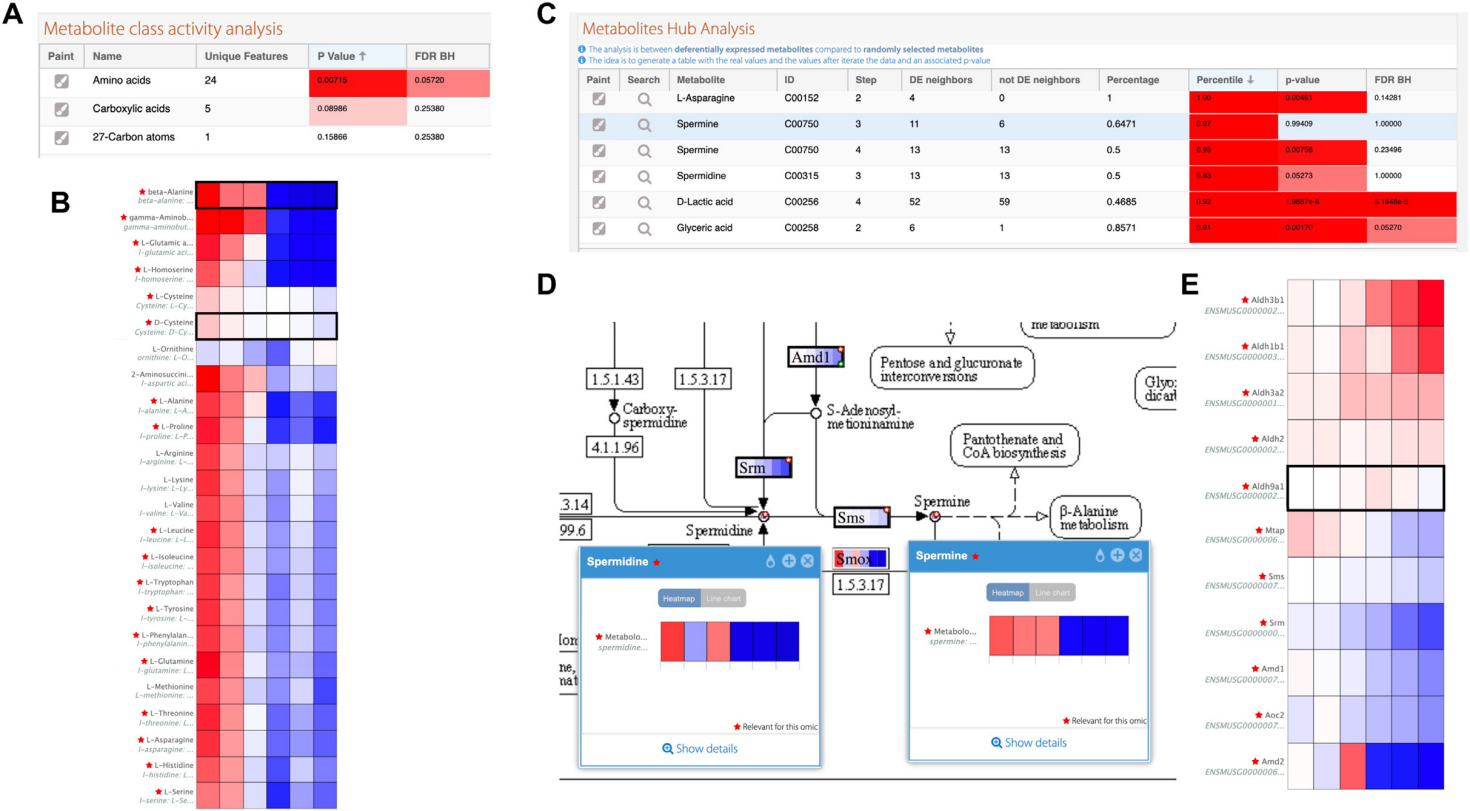
Metabolite class and hub analyses of STATegra data. (**A**) PaintOmics 4 panel with Metabolite Class Activity analysis results. (**B**) Heatmap of amino-acid values during B3 cell differentiation. (**C**) PaintOmics 4 panel with Metabolite Hub Analysis results. (**D**) Neighbouring network for spermine and spermidine in KEGG. (**E**) Expression values for spermine neighbouring differentially expressed genes.

Pathway enrichment analysis based on metabolomics data did not detect any significant pathways, possibly due to the limited number of metabolites in this dataset. However, the Metabolite Class Activity analysis identified amino acids as significant and, marginally, also carboxylic acids (Figure 3A). Accordingly, most amino acids had higher values at early time points (Figure 3B), which is consistent with the high proliferative state of the pre-BI stage where protein synthesis is highly active (38). Moreover, the metabolite hub analysis of the STATegra data highlighted a number of compounds as having a high proportion of DEG in their proximal network, among them three polyamines: spermidine, putrescine, and spermine (Figure 3C). These metabolites have higher levels at the pre-BI stage and decrease as cells differentiate towards pre-BII (Figure 3D). Neighbouring genes for these metabolites included Srm (Spermidine synthase), Sms (Spermine synthase) and Amd1 (*S*-adenosylmethionine decarboxylase proenzyme 1) which were downregulated during differentiation (Figure 3E). Ikaros triggers pre-B-cell differentiation through repression of the c-Myc transcription factor (39), which is known to regulate the expression of polyamine synthesis genes such as Srm, Sms and Amd1 genes (40). Therefore, repression of c-Myc is consistent with the observed downregulation of these genes and of the three polyamines in the STATegra dataset. Moreover, spermidine, putrescine, and spermine have an established role in cell proliferation (41) and are likely to play a key role in T-cell and B-cell differentiation (42). The PaintOmics 4 analysis highlights the polyamine metabolite/gene regulatory hub during the murine B3 cell differentiation course.

### PaintOmics 4 analysis of *Arabidopsis* drought response leverages MapMan and KEGG pathways for novel pathway insights

We used the *Arabidopsis* BRL3ox study (31) to showcase the utility of PaintOmics 4 for the interpretation of multi-omics data from plants. This study evaluated the response to drought conditions of a mutant overexpressing BRL3, a plant brassinosteroid receptor. Roots RNA-seq and metabolomics data after 5 days of drought treatment, together with a list of differentially expressed features, were available.

**Figure 4.**
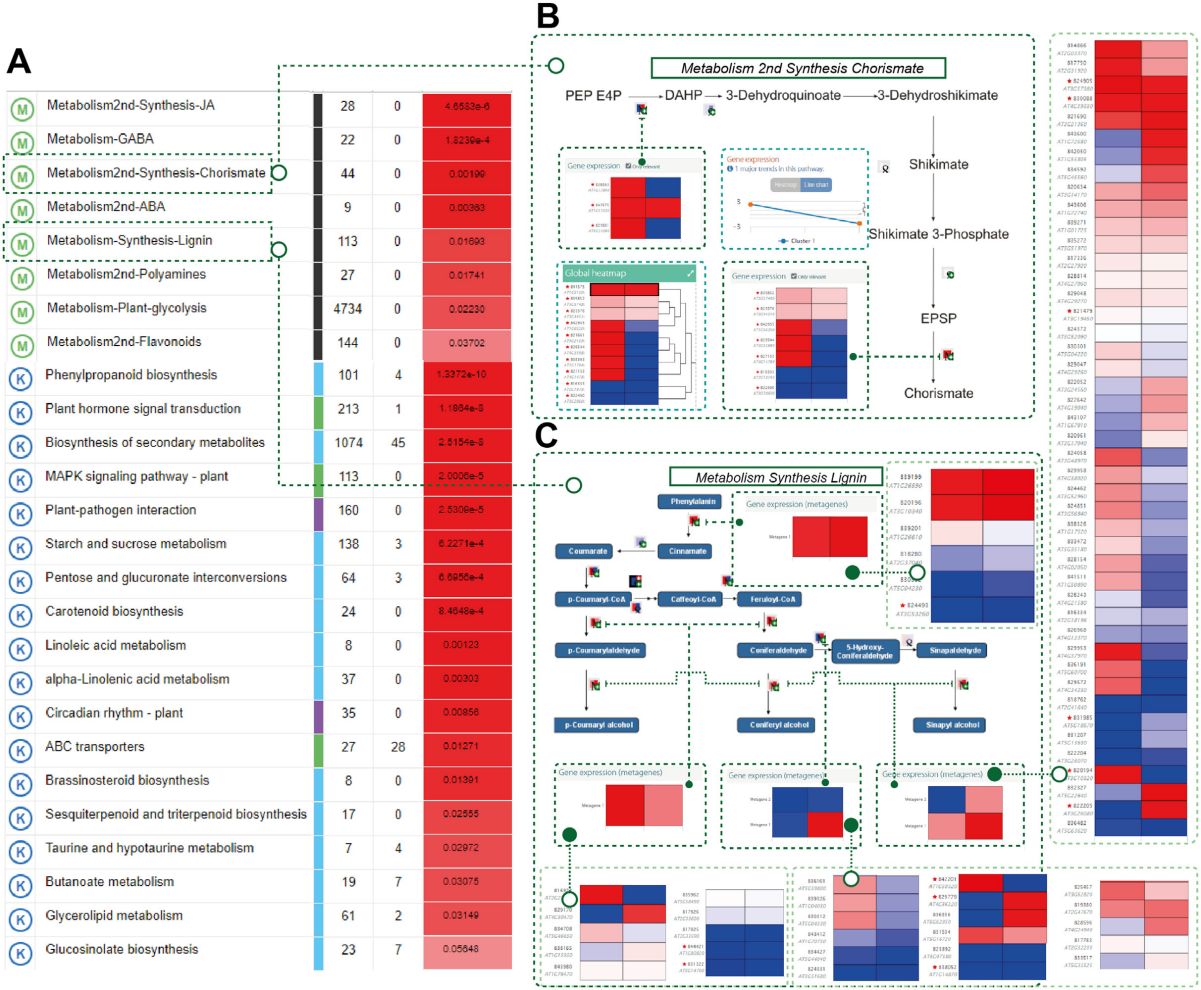
PaintOmics 4 analysis of Arabidopsis BRL3ox data. (**A**) List of enriched pathways. (**B**) MapMan synthesis chorismate pathway. The pathway shows a downregulation trend at 5 days. (**C**) MapMan synthesis lignin pathway. The pathway contains a large number of BINs with many associated genes. The figure shows both metagene representations and the heatmaps of the corresponding genes linked by green connectors.

We run PaintOmics 4 on the BRL3ox data using the KEGG and MapMan databases. While KEGG is a general pathway database, MapMan is tailored to plants and contains a more detailed representation of plant-specific pathways. A total of 18 and 8 enriched pathways were found for KEGG and MapMan databases, respectively (combined adjusted *P*-value < 0.05) (Figure 4A, Supplementary Table S2). KEGG results indicated enrichment of multiple metabolic pathways (e.g. Phenylpropanoid biosynthesis, Biosynthesis of secondary metabolites, Brassinosteroid metabolism, among others), as well as signalling pathways, including MAPK signalling pathway, ABC transporters and the general Plant hormone signal transduction. MapMan, however, returned an enrichment picture that highlighted the role of specific hormones (synthesis of jasmonic acid, GABA, abscisic acid), secondary metabolites (flavonoids, chorismate, polyamines) and the synthesis of lignin in the BLR3ox response to drought, complementing the biological interpretation provided by KEGG. Many of these processes were discussed in the original publication, supporting the robustness of PaintOmics 4 analysis. Here, we discussed two novel results not identified in the previous study.

The Synthesis of Chorismate pathway was found enriched by the PaintOmics 4 MapMan analysis. Visual inspection of this pathway indicated a general downregulation in the BRL3ox mutant under drought conditions (Figure 4B). This pathway catalyses the formation of chorismate, the last step in the shikimate pathway and a branch-point metabolite used for the synthesis of aromatic amino acids, p-aminobenzoic acid, folate, and other cyclic metabolites such as ubiquinone (43). Under abiotic stress conditions, plants activate the synthesis of aromatic compounds through the shikimate pathway, improving salt stress tolerance but not causing oxidative or drought stress (44). However, some aromatic compounds, such as m-tyrosine, inhibit the growth of many plant species by slowing down root development, and high tryptophan levels have been reported to inhibit root growth (45). Interestingly, both tyrosine and tryptophan were down regulated in BRL3ox plants compared to the WT plants under drought conditions (31). We speculate that BRL3ox overexpression results in downregulation of chorismate pathway and aromatic compound synthesis under drought, improving drought tolerance without growth arrest.

Another significant MapMan pathway was synthesis of lignin, which shows upregulation in treated BRL3ox plants compared to the WT (Figure 4C). This pathway is represented in the MapMan database by 113 different genes distributed into 12 reactions, which implies that multiple steps in the pathways are associated with a large number of genes. For example, the last steps of the pathways represent the conversion from aldehyde to alcohol of coumaryl, coniferyl and synapil, catalysed by cinnamyl alcohol dehydrogenase protein family, with 44 associated genes, which would be difficult to include in the map. The PaintOmics metagene function calculated two major upregulating trends for this node, providing a more interpretable representation for the reaction (Figure 4C). Lignin is known to play an important role in improving plants’ drought resistance through water transport and mechanical support (46). The PaintOmics 4 analysis suggests that the coordinated upregulation of gene families catalysing monolignol synthesis is part of the BRL3ox mechanism of drought resistance.

## DISCUSSION

While multi-omics studies have increased in number, scope, sample size and diversity of measured omics modalities, and a wide range of integrative statistical data analysis tools have been proposed, the biological interpretation of these data is still a major challenge. PaintOmics 4 addresses this problem by projecting processed values of multi-omics features onto pathway maps. However, the success of a pathway analysis strategy depends on the amount and identity of the features captured in the database and on their distribution among pathway definitions. Different biological pathway tools focus on different types of organisms, cellular processes, and types of reactions, thereby offering different but complementary views of the biology. Our use cases showed that, by implementing different databases in PaintOmics 4, complementary information about the experiment is gained, and interpretation of the data is improved. To the best of our knowledge, PaintOmics 4 is the only tool to combine these three pathway databases under one analysis.

Another distinctive aspect of PaintOmics 4 is its versatility in representing virtually any omics modality on pathways. This implies that omics layers that act as direct or indirect regulators of pathway activities, such as epigenetic marks or microRNAs, can be interpreted from the perspective of the pathways that they impact. PaintOmics 4 not only provides enrichment analysis for these layers, but further improves interpretability by showing the relationship between the regulatory feature and regulated gene on the pathway maps. Importantly, and in contrast to other methods that do accept regulatory omics data for pathway views (21) PaintOmics 4 displays multiple regulatory information simultaneously on the pathway representation both globally and for each pathway node, allowing for different levels of granularity in the analysis. This unique functionality implies that both *cis* and *trans* regulatory relationships can be directly linked to the mechanistic representation of the biological process captured by the pathway, thereby facilitating the understanding of the multi-layer component of the multi-omics study.

Analysis and interpretation of metabolomics experiments are particularly challenging because usually pathways only accurately represent a fraction of the measured metabolites (e.g. in lipidomics, many different compounds might be represented by the same entity in the pathway) and/or, many metabolites present in the metabolic network are not measured in the metabolomics experiment. Moreover, *a priori* hypotheses of metabolite relevance for the study may dictate the type of metabolomics assay to be run (our STATegra and BLR3ox use cases are examples of this). In such a case, a large fraction of the measured compounds may show significant changes, and this jeopardises any enrichment analysis strategy. To still be able to evaluate if the targeted metabolite types are affected in the experiment, we introduced the Metabolite Class Activity analysis, where we test the hypothesis of the measured metabolite class having a high proportion of compounds with significant changes. Another type of question that multi-omics studies involving metabolomics may pose is the link between the metabolite change and the regulation of the expression of the genes, proteins or other compounds that could modify their metabolite levels. This is relevant, for example, when looking for metabolite biomarkers or targets of metabolic control. This question can be addressed by a gene-metabolite bipartite network analysis (47) or by a flux balance analysis (FBA) strategy (48). However, gene-metabolite correlation networks usually lack the pathway context and FBA returns information on fluxes rather than compounds, requires a complex mathematical formulation, and still has limited adaptations to multi-omics data (49). In PaintOmics 4, we propose a simple approach based on the analysis of the local metabolite network to identify the proportion of differentially expressed features. Applied to our STATegra dataset, this method identified a number of metabolites displaying hub-like properties (spermidine, spermine, citric-acid, etc.) and complemented the limited metabolomics enrichment analysis results. As the hub analysis panel also provides links to the involved features and pathways, the user has all the information on hand to navigate and interpret the data.

In summary, PaintOmics 4 is a web server for the multi-layered biological interpretation of multi-omics data that includes a wealth of resources for a comprehensive and interactive analysis. Future developments will address the growing applications of the multi-omics paradigm to assist precision medicine and single-cell analyses.

## DATA AVAILABILITY

PaintOmics 4 web server is available at https://paintomics.org/. PaintOmics 4 code and documentation are available at https://github.com/ConesaLab/paintomics4.

## Supplementary Material

gkac352_Supplemental_Files

## References

[1] National Institutes of Health NHLBI trans-omics for precision medicine. 2022; https://www.nhlbi.nih.gov/science/trans-omics-precision-medicine-topmed-program.

[2] Cancemi P. , Buttacavoli M., Di Cara G., Albanese N.N., Bivona S., Pucci-Minafra I., Feo1 S. A multiomics analysis of S100 protein family in breast cancer. Oncotarget. 2018; 9:29064–29081. 30018736 10.18632/oncotarget.25561 PMC6044374

[3] Stare T. , Ramšak Ž., Križnik M., Gruden K. Multiomics analysis of tolerant interaction of potato with potato virus Y. Sci. Data. 2019; 6:250. 31673114 10.1038/s41597-019-0216-1 PMC6823367

[4] Gomez-Cabrero D. , Tarazona S., Ferreirós-Vidal I., Ramirez R.N., Company C., Schmidt A., Reijmers T., von Saint Paul V., Marabita F., Rodríguez-Ubreva J. et al. STATegra, A comprehensive multi-omics dataset of B-cell differentiation in mouse. Sci. Data. 2019; 6:256. 31672995 10.1038/s41597-019-0202-7 PMC6823427

[5] Ciriello G. , Miller M., Aksoy B., Senbabaoglu Y., Schultz N., Sander C. Emerging landscape of oncogenic signatures across human cancers. Nat. Genet. 2013; 45:1127–1133. 24071851 10.1038/ng.2762 PMC4320046

[6] Stunnenberg H. , Hirst M. The international human epigenome consortium: A blueprint for scientific collaboration and discovery. Cell. 2016; 167:1897. 10.1016/j.cell.2016.11.007 27863232

[7] Bersanelli M. , Mosca E., Remondini D., Giampieri E., Sala C., Castellani G., Milanes L. Methods for the integration of multi-omics data: mathematical aspects. BMC Bioinformatics. 2016; 17:S15. 10.1186/s12859-015-0857-9 PMC4959355 26821531

[8] Meng C. , Zeleznik O., Thallinger G., Kuster B., Gholami A., Culhane A. Dimension reduction techniques for the integrative analysis of multi-omics data. Brief. Bioinform. 2016; 17:628–641. 26969681 10.1093/bib/bbv108 PMC4945831

[9] Tarazona S. , Arzalluz-Luque A., Conesa A. Undisclosed, unmet and neglected challenges in multi-omics studies. Nat. Comput. Sci. 2021; 1:395–402. 10.1038/s43588-021-00086-z 38217236

[10] Subramanian A. , Tamayo P., Mootha V., Mukherjee S., Ebert B.L., Gillette M.A., Paulovich A., Pomeroy S.L., Golub T.R., Lander E.S. et al. Gene set enrichment analysis: A knowledge-based approach for interpreting genome-wide expression profiles. Proc. Nat. Acad. Sci. U.S.A. 2005; 102:15545–15550. 10.1073/pnas.0506580102 PMC1239896 16199517

[11] Hong M. , Pawitan Y., Magnusson P., Prince J. Strategies and issues in the detection of pathway enrichment in genome-wide association studies. Hum. Genet. 2009; 126:289–301. 19408013 10.1007/s00439-009-0676-z PMC2865249

[12] Garcia-Garcia F. , Panadero J., Dopazo J., Montaner D. Integrated gene set analysis for microRNA studies. Bioinformatics. 2016; 32:2809–2816. 27324197 10.1093/bioinformatics/btw334 PMC5018374

[13] Maksimovic J. , Oshlack A., Phipson B. Gene set enrichment analysis for genome-wide DNA methylation data. Genome Biol. 2021; 22:173–173. 34103055 10.1186/s13059-021-02388-x PMC8186068

[14] Diego R. , Tarazona S., Martínez-Mira C., Balzano-Nogueira L., Furió-Tarí P., Pappas J., Conesa A. PaintOmics 3: a web resource for the pathway analysis and visualization of multi-omics data. Nucleic Acids Res. 2018; 46:W503–W509. 29800320 10.1093/nar/gky466 PMC6030972

[15] Canzler S. , Hackermüller J. multiGSEA: a GSEA-based pathway enrichment analysis for multi-omics data. BMC Bioinformatics. 2020; 21:561–561. 33287694 10.1186/s12859-020-03910-x PMC7720482

[16] Paczkowska M. , Barenboim J., Sintupisut N., Fox N.S., Zhu H., Abd-Rabbo D., Mee M.W., Boutros P.C., Reimand J. PCAWG Drivers and Functional Interpretation Working Group et al. Integrative pathway enrichment analysis of multivariate omics data. Nat. Commun. 2020; 11:735. 32024846 10.1038/s41467-019-13983-9 PMC7002665

[17] Pavlopoulos G. , Kontou P., Pavlopoulou A., Bouyioukos C., Markou E., Bagos P. Bipartite graphs in systems biology and medicine: a survey of methods and applications. GigaScience. 2018; 7:giy014. 10.1093/gigascience/giy014 PMC6333914 29648623

[18] Shannon P. , Markiel A., Ozier O., Baliga1 N.S., Wang J.T., Ramage D., Amin N., Schwikowski1 B., Ideker T. Cytoscape: a software environment for integrated models of biomolecular interaction networks. Genome Res. 2003; 13:2498–2504. 14597658 10.1101/gr.1239303 PMC403769

[19] Kuo T. , Tian T., Tseng Y. 3Omics: a web-based systems biology tool for analysis, integration and visualization of human transcriptomic, proteomic and metabolomic data. BMC Syst. Biol. 2013; 7:64. 23875761 10.1186/1752-0509-7-64 PMC3723580

[20] Hu Z. Using VisANT to analyze networks. Curr. Prot. Bioinformatics. 2014; 45:8.8.1–8.8.39. 10.1002/0471250953.bi0808s45 PMC4240741 25422679

[21] Zhou G. , Ewald J., Xia J. OmicsAnalyst: a comprehensive web-based platform for visual analytics of multi-omics data. Nucleic Acids Res. 2021; 49:W476–W482. 34019646 10.1093/nar/gkab394 PMC8262745

[22] Ghosh S. , Datta A., Choi H. multiSLIDE is a web server for exploring connected elements of biological pathways in multi-omics data. Nat. Commun. 2021; 12:2279. 33863886 10.1038/s41467-021-22650-x PMC8052434

[23] Sakurai N. , Ara T., Ogata Y., Sano R., Ohno T., Sugiyama K., Hiruta A., Yamazaki K., Yano K., Aoki K. KaPPA-View4: a metabolic pathway database for representation and analysis of correlation networks of gene co-expression and metabolite co-accumulation and omics data. Nucleic Acids Res. 2011; 39:D677–D684. 21097783 10.1093/nar/gkq989 PMC3013809

[24] Schwacke R. , Ponce-Soto G., Krause K., Bolger A.M., Arsova1 B., Hallab1 A., Gruden K., Stitt M., Bolger1 M.E., Usadel B. et al. MapMan4: A refined protein classification and annotation framework applicable to multi-omics data analysis. Mol. Plant. 2019; 12:879–892. 30639314 10.1016/j.molp.2019.01.003

[25] Conesa A. , Hernández R. Omics data integration in systems biology. 2014; 64:441–459.

[26] Molenaar M. , Jeucken A., Wassenaar T., Lest C., Brouwers J., Helms J. LION/web: a web-based ontology enrichment tool for lipidomic data analysis. GigaScience. 2019; 8:giz061. 31141612 10.1093/gigascience/giz061 PMC6541037

[27] Garcia-Alcalde F. , Garcia-Lopez F., Dopazo J., Conesa A. Paintomics: a web based tool for the joint visualization of transcriptomics and metabolomics data. Bioinformatics. 2011; 27:137–139. 21098431 10.1093/bioinformatics/btq594 PMC3008637

[28] Haw R. , Hermjakob H., D’Eustachio P., Stein L. Reactome pathway analysis to enrich biological discovery in proteomics datasets. Proteomics. 2011; 11:3598–3613. 21751369 10.1002/pmic.201100066 PMC4617659

[29] Benjamini Y. , Hochberg Y. Controlling the false discovery rate: a practical and powerful approach to multiple testing. J. R. Stat. Soc. Ser B. 1995; 57:289–300.

[30] Ponzoni I. , Nueda M., Tarazona S., Götz S., Montaner D., Dussaut J.S., Dopazo J., Conesa A. Pathway network inference from gene expression data. BMC Syst. Biol. 2014; 8:S7. 10.1186/1752-0509-8-S2-S7 PMC4101702 25032889

[31] Fàbregas N. , Lozano-Elena F., Blasco-Escámez D., Tohge T., Martínez-Andújar C., Albacete A., Osorio S., Bustamante M., Riechmann J.L., Nomura T. et al. Overexpression of the vascular brassinosteroid receptor BRL3 confers drought resistance without penalizing plant growth. Nat. Commun. 2018; 9:4680. 30409967 10.1038/s41467-018-06861-3 PMC6224425

[32] Mosteller F. , Fisher R. Questions and answers. Am. Stat. 1948; 2:30–31.

[33] Ferreirós-Vidal I. , Carroll T., Zhang T., Lagani V., Ramirez R.N., Ing-Simmons E., Gómez-Valadés A.G., Cooper L., Liang Z., Papoutsoglou G. et al. Feedforward regulation of Myc coordinates lineage-specific with housekeeping gene expression during B cell progenitor cell differentiation. PLOS Biol. 2019; 17:e2006506. 30978178 10.1371/journal.pbio.2006506 PMC6481923

[34] Pachnis V. , Mankoo B., Costantini F. Expression of the c-RET proto-oncogene during mouse embryogenesis. Development (Cambridge, England). 1994; 119:1005–1017. 10.1242/dev.119.4.1005 8306871

[35] Rusmini M. , Griseri P., Lantieri F., Matera I., Hudspeth K.L., Roberto A., Mikulak J., Avanzini S., Rossi V., Mattioli G. et al. Induction of RET dependent and independent pro-inflammatory programs in human peripheral blood mononuclear cells from Hirschsprung patients. PLoS one. 2013; 8:e59066. 23527089 10.1371/journal.pone.0059066 PMC3601093

[36] Darnell James E. , Kerr lan M., Stark George R. Jak-STAT pathways and transcriptional activation in response to IFNs and other extracellular signaling proteins. Science. 1994; 264:1415–1421. 8197455 10.1126/science.8197455

[37] Pichler M. , Stiegelbauer V., Vychytilova-Faltejskova P., Ivan C., Ling H., Winter E., Zhang X., Goblirsch M., Wulf-Goldenberg A., Ohtsuka M. et al. Genome-Wide miRNA analysis identifies miR-188-3p as a novel prognostic marker and molecular factor involved in colorectal carcinogenesis. Clin. Cancer Res. 2017; 23:1323. 27601590 10.1158/1078-0432.CCR-16-0497 PMC5544252

[38] Ferreiros-Vidal I. , Carroll T., Taylor B., Terry A., Liang Z., Bruno L., Dharmalingam G., Khadayate S., Cobb B.S., Smale S.T. et al. Genome-wide identification of Ikaros targets elucidates its contribution to mouse B-cell lineage specification and pre-B-cell differentiation. Blood. 2013; 121:1769–1782. 23303821 10.1182/blood-2012-08-450114

[39] Ma S. , Pathak S., Mandal M., Trinh L., Clark M.R., Lu R. Ikaros and aiolos inhibit pre-b-cell proliferation by directly suppressing c-myc expression. Mol. Cell. Biol. 2010; 30:4149–4158. 20566697 10.1128/MCB.00224-10 PMC2937562

[40] Guo Y. , Ye Q., Deng P., Cao Y., He D., Zhou Z., Wang C., Zaytseva Y.Y., Schwartz C.E., Lee E.Y. et al. Spermine synthase and MYC cooperate to maintain colorectal cancer cell survival by repressing Bim expression. Nat. Commun. 2020; 11:3243. 32591507 10.1038/s41467-020-17067-x PMC7320137

[41] Li J. , Meng Y., Wu X., Sun Y. Polyamines and related signaling pathways in cancer. Cancer Cell Int. 2020; 20:539. 33292222 10.1186/s12935-020-01545-9 PMC7643453

[42] Hesterberg R. , Cleveland J., Epling-Burnette P. Role of polyamines in immune cell functions. Med. Sci. (Basel, Switzerland). 2018; 6:22. 10.3390/medsci6010022 PMC5872179 29517999

[43] Fagan R. , Palfey B. 7.03 - Flavin-Dependent Enzymes. 2010; Elsevier Oxford 37–113.

[44] Oliva M. , Guy A., Galili G., Dor E., Schweitzer R., Amir R., Hacham Y. Enhanced production of aromatic amino acids in tobacco plants leads to increased phenylpropanoid metabolites and tolerance to stresses. Front Plant Sci. 2020; 11:604349. 33510749 10.3389/fpls.2020.604349 PMC7835393

[45] Staswick P. The tryptophan conjugates of jasmonic and indole-3-acetic acids are endogenous auxin inhibitors. Plant Physiol. 2009; 150:1310–1321. 19458116 10.1104/pp.109.138529 PMC2705031

[46] Lima T. , Carvalho E., Martins F., Oliveira R., Miranda R., Müller C., Pereira L., Bittencourt P., Sobczak J., Gomes-Filho E. et al. Lignin composition is related to xylem embolism resistance and leaf life span in trees in a tropical semiarid climate. New Phytologist. 2018; 219:1252–1262. 29767841 10.1111/nph.15211

[47] Mor D. , Huertas F., Morse A., Kaletsky R., Murphy C., Kalia V., Miller G.W., Moskalenko O., Conesa A., Mor D.E. GAIT-GM integrative cross-omics analyses reveal cholinergic defects in a *C. elegans* model of Parkinson’s disease. Sci. Rep. 2022; 12:3268. 35228596 10.1038/s41598-022-07238-9 PMC8885929

[48] Oberhardt M. , Chavali A., Papin J. Flux balance analysis: interrogating genome-scale metabolic networks. Methods Mol. Biol. 2009; 500:61–80. 19399432 10.1007/978-1-59745-525-1_3

[49] Jensen P. , Papin J. Functional integration of a metabolic network model and expression data without arbitrary thresholding. Bioinformatics. 2011; 27:541–547. 21172910 10.1093/bioinformatics/btq702 PMC6276961

