# Supplementary material for: PaintOmics 4: new tools for the integrative analysis of multi-omics datasets supported by multiple pathway databases
Source: Nucleic Acids Res. 2022 May 24;50(W1):W551–9. doi: 10.1093/nar/gkac352 (PMC9252773; doi:10.1093/nar/gkac352)
Supplement: gkac352_Supplemental_Files [file gkac352_supplemental_files.zip › SupplementaryFiguresRevision.pdf]

PaintOmics 4: New tools for the integrative analysis of multi-omics datasets supported by multiple pathway databases

Tianyuan Liu[1], Pedro Salguero[2], Marko Petek[3], Carlos Martinez-Mira[4], Leandro Balzano-Nogueira[5], Živa Ramšak[3], Lauren McIntyre[6], Kristina Gruden[3], Sonia Tarazona[2], Ana Conesa[7,8*]
[1] Department of Engineering, Cardiff University, Cardiff, United Kingdom
[2] Department of Statistics, Operation Research and Quality, Universitat Politècnica de València, Valencia, Spain
[3] Department of Biotechnology and Systems Biology, National Institute of Biology, Ljubljana, Slovenia
[4] Biobam Bioinformatics, Valencia, Spain
[5] Diabetes Institute, University of Florida, Gainesville, USA
[6] Department of Molecular Genetics and Microbiology, Genetics Institute, University of Florida, Gainesville, USA
[7] Institute for Integrative Systems Biology, Spanish National Research Council (CSIC), Paterna, Spain
[8] Department of Microbiology and Cell Science, University of Florida, Gainesville, USA

* To whom correspondence should be addressed. Tel: +34963544771 Email: ana.conesa@csic.es

Supplementary Figures

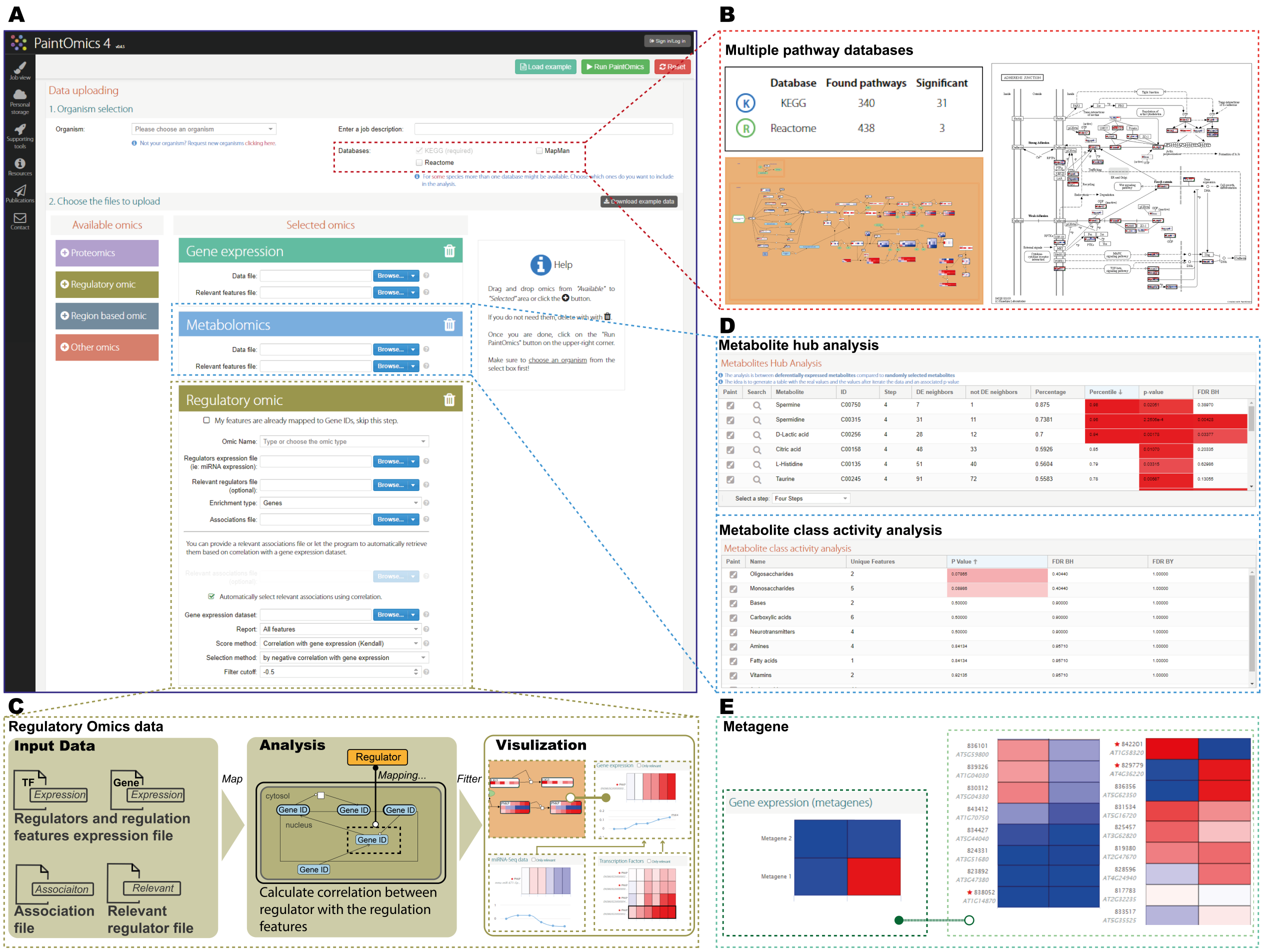

Supplementary Figure 1. Schematic representation of improvements in PaintOmics 4. (A) The main user interfaces with showing multiple options for database selection and the novel Regulatory Omic data input panel. PaintOmics 4 supports a diversity of pathway databases and virtually any omics modality where features can be mapped to gene, protein, or metabolite. (B) Multiple pathway databases can be analysed simultaneously. Multi-omics data are simultaneously analysed in the selected databases. (C) Analysis workflow for Regulatory Omic data. PaintOmics 4 expands the Regulatory Omics data input to deal with trans-regulatory relationships. On this panel, expression values for genes and regulators can be uploaded, together with an annotation file of user-defined associations. Additionally, a correlation threshold value can be provided to limit associations to those of a certain magnitude. This information is used to map regulators to pathways at the positions of their targeted genes and to compute pathway enrichment based either on the number of significant regulators mapping to each pathway, or the number of regulated genes. (D) Metabolite class activity and Metabolite hub analyses interfaces. This analysis identifies which pathways are impacted by the regulatory layer. Moreover, PaintOmics 4 implements novel methods to analyse and integrate metabolomics data. The metabolite Hub Analysis detects compounds that have a high accumulation of differentially expressed genes in their proximity within the metabolic network, while the Metabolite Class Activity analysis tests the hypothesis of a given metabolic class having a proportion of significant metabolites higher than a user-provided threshold. (E) Metagene representations of a multi-feature mode with two expression trends. Metagenes, a representation of the various trends of a large number of features, is generalised to summarise omics data of pathways and pathway nodes with multiple associated genes.

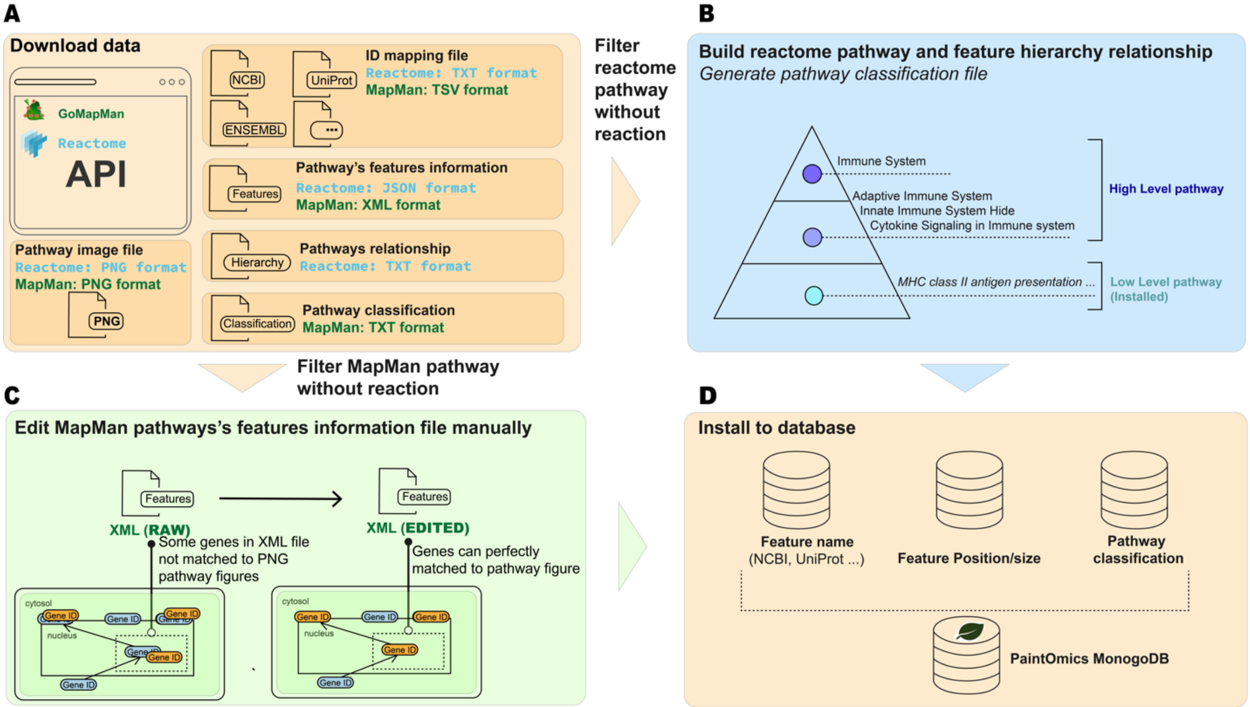

Supplementary Figure 2. Installation of Reactome and MapMan databases into PaintOmics 4. (A) Data files containing ID mapping data, Pathway features, Pathway classification or Pathway relationships were downloaded from the Reactome and MapMan sites. (B) Reactome Pathway relationships were assimilated to Pathway categories and only low-level pathways, containing features and reactions were integrated. (C) Edition of MapMan xml files to match node coordinates in pathway images. (D) New pathway data was integrated in the PaintOmics Mongo database. Only pathways containing reactions were added. An extra "database source" field was added to Mongo and other PaintOmics data structures.

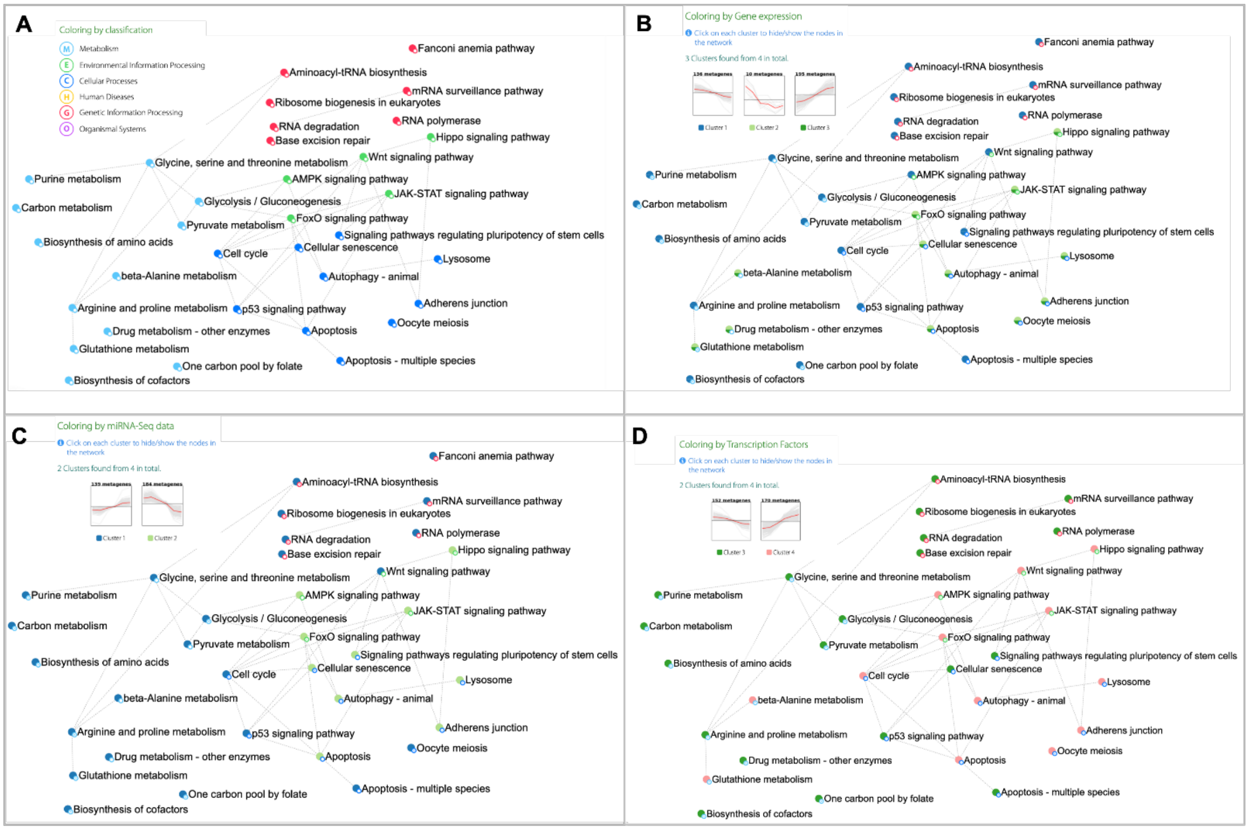

Supplementary Figure 3. STATegra multi-layered networks. (A) KEGG pathway network of enriched pathways coloured by KEGG category. Links indicate shared genes between pathways. The pathway network was created based on the shared features of the significant KEGG pathways. PaintOmics calculates pathway metagenes describing pathway variation trends for each omics modality, cluster similar metagenes and colour them in the network by cluster assignment. This results in a coloured network per omics type that represents how pathways are regulated at each molecular layer. (B) Same network as in (A) but coloured according to RNA-seq metagene clustering. For gene expression, most metabolic and genetic information pathways were downregulated, while signalling pathways showed both up and down regulation trends. (C) Same network as in (A) but coloured according to microRNA-seq metagene clustering. microRNAs associated genes in these pathways tended to be upregulated at late-time points (pre-BII stage) in metabolic and genetic information processing pathways, while upregulation for signalling pathways took place at early time points (pre-BI stage). (D) Same network as in (A) but coloured according to Transcription Factor (TF) metagene clustering. TF regulation showed the opposite behaviour, with TFs that bind metabolic and genetic information processing pathways being downregulated as differentiation progresses but upregulated for signalling pathway genes.
